# Individual differences in physiologic measures are stable across repeated exposures to total sleep deprivation

**DOI:** 10.14814/phy2.12129

**Published:** 2014-09-28

**Authors:** Eric Chern‐Pin Chua, Sing‐Chen Yeo, Ivan Tian‐Guang Lee, Luuan‐Chin Tan, Pauline Lau, Sara S. Tan, Ivan Ho Mien, Joshua J. Gooley

**Affiliations:** 1 Program in Neuroscience and Behavioral Disorders, Duke‐NUS Graduate Medical School Singapore, Singapore, 169857, Singapore; 2 National Neuroscience Institute, Singapore, 308433, Singapore; 3 Graduate School for Integrative Sciences and Engineering, National University of Singapore, Singapore, 117456, Singapore

**Keywords:** circadian, EEG, performance, sleep

## Abstract

Some individuals show severe cognitive impairment when sleep deprived, whereas others are able to maintain a high level of performance. Such differences are stable and trait‐like, but it is not clear whether these findings generalize to physiologic responses to sleep loss. Here, we analyzed individual differences in behavioral and physiologic measures in healthy ethnic‐Chinese male volunteers (*n* = 12; aged 22–30 years) who were kept awake for at least 26 h in a controlled laboratory environment on two separate occasions. Every 2 h, sustained attention performance was assessed using a 10‐min psychomotor vigilance task (PVT), and sleepiness was estimated objectively by determining percentage eyelid closure over the pupil over time (PERCLOS) and blink rate. Between‐subject differences in heart rate and its variability, and electroencephalogram (EEG) spectral power were also analyzed during each PVT. To assess stability of individual differences, intraclass correlation coefficients (ICC) were determined using variance components analysis. Consistent with previous work, individual differences in PVT performance were reproducible across study visits, as were baseline sleep measures prior to sleep deprivation. In addition, stable individual differences were observed during sleep deprivation for PERCLOS, blink rate, heart rate and its variability, and EEG spectral power in the alpha frequency band, even after adjusting for baseline differences in these measures (range, ICC = 0.67–0.91). These findings establish that changes in ocular, ECG, and EEG signals are highly reproducible across a night of sleep deprivation, hence raising the possibility that, similar to behavioral measures, physiologic responses to sleep loss are trait‐like.

## Abstract (precis)

e12129

Individual differences in physiologic measures were examined in healthy ethnic‐Chinese males who underwent sleep deprivation in the laboratory on two different occasions. We found that between‐subject differences in ocular, electrocardiogram, and electroencephalogram measures were highly stable, even after adjusting for baseline individual differences in these measures. These results suggest that the brain responds predictably to the challenge of sleep deprivation and raise the possibility that physiologic responses to sleep loss are trait‐like.

## Introduction

Sleep deprivation impairs neurobehavioral performance and increases risk of occupational and motor vehicle accidents (Lyznicki et al. 1998; Basner et al. 2013). The cognitive effects of sleep loss differ substantially between persons, however, with some individuals displaying marked functional impairment and others showing only modest changes from baseline. Such individual differences in neurobehavioral performance show trait‐like stability across repeated exposures to sleep deprivation (Van Dongen et al. 2004; Rupp et al. 2012). That is, within‐subject variability is much smaller compared to between‐subject differences, suggesting a stable neurobiological basis for cognitive responses to sleep loss. This view is supported by functional magnetic resonance imaging (fMRI) studies demonstrating that task‐dependent changes in brain activation during a working memory task are stable across exposures to total sleep deprivation (Lim et al. 2007). More recently, it was shown that sustained attention performance during sleep deprivation has a strong heritable component, as assessed in monozygotic versus dizygotic twin pairs (Kuna et al. 2012). These studies suggest that genetic factors play an important role in determining a person's cognitive vulnerability to sleep deficiency.

In addition to inducing neurobehavioral impairment, total sleep deprivation is associated with changes in physiologic signals including ocular, electrocardiogram (ECG), and electroencephalogram (EEG)‐derived measures, some of which are used to assess sleepiness objectively. For example, as wakefulness is extended beyond usual bedtime, short‐duration blinks are supplanted by longer duration eye closure events and slow eye movements (Cajochen et al. 1999; Chua et al. 2012). Concurrently, EEG spectral power increases in delta and theta frequency bands (Borbely et al. 1981; Finelli et al. 2000), and changes in heart rate variability parallel the time‐course of lapses in vigilance (Chua et al. 2012). Brain activation patterns also differ markedly between rested and sleep‐deprived states, including changes in functional network connectivity during task performance (De Havas et al. 2012). In addition to its effects on objective sleepiness and brain activity, exposure to sleep deprivation impairs glucose tolerance (Spiegel et al. 1999), increases inflammatory markers associated with higher cardiovascular risk (Mullington et al. 2009), and elevates systolic blood pressure in response to acute psychological stress (Franzen et al. 2011), suggesting an important role for sleep in cardiometabolic health. These studies demonstrate that sleep loss impacts a broad range of physiologic measures.

To date, physiologic responses to sleep deprivation have been studied primarily at the group level, even though it is widely recognized that there is marked interindividual variation. As such, it is not known whether between‐subject differences in physiologic measures are stable and reproducible across repeated exposures to sleep deprivation. The focus of the present study was to determine the extent to which individual differences in ocular, ECG, and EEG measures are reproducible during prolonged wakefulness. Here, we demonstrate that between‐subject differences in vigilance and physiologic measures were highly stable in individuals who were twice exposed to total sleep deprivation in a controlled laboratory setting.

## Materials and Methods

### Ethical approval

Written informed consent was obtained from all participants, and research procedures were approved by the SingHealth Centralized Institutional Review Board. Procedures were compliant with ethical principles for medical research described in the Declaration of Helsinki.

### Subjects

Healthy ethnic‐Chinese males (*n* = 12) aged 22–30 years were recruited from the general population. Health was assessed using a structured questionnaire. Participants reported no use of medications or nicotine products. Definite morning types and evening types were excluded using the Horne‐Östberg morningness–eveningness questionnaire (MEQ score <31 or >69) (Horne and Ostberg 1976). Only participants who reported good quality sleep were eligible, assessed using the Pittsburgh Sleep Quality Index (PSQI ≤5) (Buysse et al. 1989). Subjects were ineligible if they worked night shifts (between 11 pm to 7 am) or if they traveled across time zones within 3 weeks prior to the start of each study. Prior to each laboratory visit, participants were required to keep a fixed daily sleep–wake schedule for at least 1 week with 8 h of time in bed for sleep at night, and this was verified by actigraphy monitoring (Actiwatch‐L or Actiwatch 2, MiniMitter, Inc., Bend, OR). Subjects also agreed to avoid caffeine, alcohol, and over‐the‐counter medications in the week before each laboratory study.

### Study procedures

In a retrospective study, we examined behavioral and physiologic measures in subjects who completed two sleep deprivation protocols at the Chronobiology and Sleep Laboratory (CSL), Duke‐NUS Graduate Medical School Singapore. In one study, participants were kept awake for 26 h (Protocol 1) (Ho Mien et al. 2014), whereas in the other study participants were kept awake for 40 h (Protocol 2) (Chua et al. 2012). Here, we only analyzed data that were common to both protocols, that is, across 26 h of sustained wakefulness. Although not planned, the order of Protocol 1 and Protocol 2 was balanced across subjects, and study visits were separated by at least 75 days (range, 2.5–15 months).

During each laboratory study, subjects lived individually in a research suite without windows or access to time cues. Participants arrived in the evening and went to bed at their regular prestudy sleep time. Subjects were given 8 h of time in bed for sleep in darkness. If subjects awoke spontaneously before their scheduled wake time, they remained in bed in darkness until the end of the sleep opportunity. Subjects then underwent at least 26 h of prolonged wakefulness using constant routine (CR) procedures (Duffy and Dijk 2002). During the CR procedure, the head of the bed was raised to a 45° angle to place participants in a semirecumbent position, and ambient lighting was kept dim (<5 lux measured at eye level) to avoid light‐induced resetting of circadian rhythms. In addition, participants were given identical snacks every hour consisting of a small portion of granola and mixed berry juice. Researchers were present at all times to carry out the sleep deprivation protocol and to ensure subject compliance.

### Self‐rated sleepiness and sustained attention performance

Every hour, subjects rated their sleepiness on a visual analogue scale (VAS) by selecting a point on a line that was labeled with the word pair “sleepy” and “alert” at opposite ends. Every 2 hours, participants completed a 10‐min Psychomotor Vigilance Task (PVT), which is a reaction time test used to assess sustained visual attention. During the PVT, participants were asked to respond as quickly as possible to a simple visual stimulus presented at random interstimulus intervals (1 ms resolution) ranging from 2–10 sec (Dinges and Powell 1985). PVT lapses were defined as response times that exceeded 0.5 sec. The VAS and PVT were administered by computer using E‐Prime 2 Professional software (Psychology Software Tools, Inc., Sharpsburg, PA). Tasks were presented on an LCD monitor placed on an over‐bed table, hence allowing participants to complete the tasks while remaining in bed during the CR procedure.

### Physiologic measurements

#### Polysomnography

Polysomnographic recordings were performed during baseline sleep and the CR procedure. Electrodes were placed on the scalp according to the standard international 10–20 system of electrode placement. During sleep, the EEG was recorded from central (C3–A2, C4–A1) and occipital (O1–A2, O2–A1) derivations; the EOG was recorded from electrodes placed lateral to and slightly above (right) and below (left) each eye; the EMG was recorded with electrodes placed on the chin and submentally; and the ECG was recorded using a modified lead V5 configuration with electrodes placed just below the clavicle on the right shoulder and below the fifth intercostal space at the anterior axillary line. The waking EEG was recorded from the z‐line using frontal (Fz), central (Cz), parietal (Pz), and occipital (Oz) derivations referenced to the mastoids (A1 and A2), and the EOG and ECG were recorded using the same procedures described for the sleep montage. All signals were bandpass‐filtered online (EEG, EOG, and ECG at 0.3–35 Hz, EMG at 10–100 Hz), and recorded at 200 Hz using a Comet Portable EEG system (Astro‐Med, Inc., West Warwick, RI).

#### Eyelid closure monitoring

Infrared pupillography was performed during the PVT to assess percentage eyelid closure over the pupil over time (PERCLOS). Pupil diameter of the left eye was recorded at 120 Hz using a head‐mounted eye tracker that was worn like a visor (ISCAN, Inc., Woburn, MA). In three subjects (Subjects D, H, I), pupillography was not performed during their first visit.

#### Body temperature monitoring

During prolonged wakefulness, core body temperature data were collected continuously using an ingestible temperature sensor (Minimitter, Inc., Bend, OR). Data were transmitted every minute to a VitalSense Integrated Physiologic Monitor placed near the subject in bed. Participants ingested the transmitter just prior to bedtime of the baseline sleep opportunity.

### Data analysis

#### Actigraphy analysis

To estimate sleep behavior in the week prior to each laboratory visit, actigraphy data were analyzed using Actiware 5 software (MiniMitter, Inc., Bend, OR). Sleep diary entries were used to mark time in bed in the actigraphy record. Sleep onset was defined as the beginning of the first 5‐min block of epochs with all but one epoch scored as immobile. Similarly, sleep offset was defined as the end of the last 5‐min block of epochs with all but 1 epoch scored as immobile. Total sleep time (TST) was defined as the total duration of scored sleep from sleep onset to sleep offset, and sleep efficiency was calculated as TST divided by time in bed for sleep. TST and sleep efficiency were then averaged across the 7 days of data collection prior to each study visit. Actigraphy data were not available for two subjects during their second visit (Subjects G, H) due to equipment failure.

#### Sleep staging and EEG spectral analysis

Sleep staging was performed using the Somnolyzer 24 × 7 system (The Siesta Group Schlafanalyse GmbH, Vienna, Austria) according to standard Rechtschaffen and Kales criteria (Anderer et al. 2005). Sleep architecture was quantified by the following polysomnographic measures: time in bed, total sleep time, sleep latency, sleep efficiency, time spent in Stage 1 (S1), Stage 2 (S2), Stages 3 and 4 (slow wave sleep, SWS), and REM sleep (in minutes and as a proportion of total sleep time), and wake after sleep onset. NREM‐REM sleep cycles were delineated as described previously (Feinberg and Floyd 1979; Aeschbach and Borbely 1993). In brief, one sleep cycle was defined as a NREM sleep episode of at least 15 min followed by a REM sleep episode of at least 5 min. NREM sleep episodes were taken from the first occurrence of S2 sleep to the first occurrence of REM sleep within a cycle, or to final awakening. By definition, REM sleep episodes therefore spanned from one NREM sleep episode to the next, and may have included occasional light NREM sleep.

EEG spectral power during sleep episodes was analyzed in running 4‐sec epochs that overlapped by 2 sec. For each epoch, EEG spectral power was estimated using FFT analysis with a Tukey window. An algorithm based on spectral power thresholds was used to exclude epochs with artifact (The Siesta Group Schlafanalyse GmbH). EEG spectral power was then log‐transformed and reduced by averaging within the first NREM sleep cycle or across the entire 8‐h sleep episode, separately for NREM sleep (S2–S4) and REM sleep. Following previous work (Aeschbach et al. 1996; Cajochen et al. 1999), spectral power was examined for slow wave activity (SWA, 0.75–4.5 Hz) and across theta (4.5–8.5 Hz), alpha (8.5–12.5 Hz), and beta (12.5–15.5 Hz) frequency bands. EEG spectral power during wakefulness was analyzed for each PVT in running 2‐sec, nonoverlapping epochs. Epochs with artifacts caused by movements, blinks, or cardiac activity were excluded. Spectral power for each epoch was estimated using the modified periodogram method (Chua et al. 2012). Data were log‐transformed and averaged across epochs within each PVT session. EEG data were then reduced by a weighted mean based on the number of artifact‐free epochs per session across rested wakefulness (six PVT sessions from 4.5 to 14.5 h after wake) and sleep deprivation (five PVT sessions from 16.5 to 24.5 h after wake).

#### Heart rate variability analysis

The RR‐interval time series was determined across each PVT session using a Hilbert transform‐based method to detect QRS peaks in the ECG recording (Benitez et al. 2001). We then determined heart rate (HR) and the standard deviation of normal‐to‐normal sinus RR intervals (SDNN) using standard methodology (Task Force of the European Society of Cardiology and the North American Society of Pacing and Electrophysiology 1996).

#### Ocular measures of sleepiness

The EOG record was scored for blinks in 2‐sec nonoverlapping epochs by a single researcher, as described previously (Chua et al. 2012). The percentage of artifact‐free epochs containing at least one blink was determined for each PVT session. Eye‐tracking data obtained during the PVT were analyzed to determine PERCLOS, defined as the percentage of time per minute that the pupil was at least 80% covered by the eyelid (Dinges et al. 1998). Given that PERCLOS is intended to measure slow eye closures, events that were shorter than 400 msec (i.e., blinks) were excluded.

#### Assessment of circadian phase

Core body temperature data were fitted with a two‐harmonic regression model with correlated noise (Brown and Czeisler 1992). The phase angle of entrainment was defined as the time difference between the minimum of the fitted rhythm and the participant's self‐selected (i.e., scheduled) wake time in the week prior to the laboratory study. The latter also corresponded to the time when the lights were turned on at the end of the baseline sleep opportunity in the laboratory, which marked the beginning of the sleep deprivation protocol.

### Statistical analysis

In each subject, data were averaged across the sleep‐deprived state (i.e., across five PVT sessions) for each study visit prior to statistical testing. Following the analysis approach of earlier work, reproducibility of individual differences in behavior and physiology was assessed using variance components analysis and quantified using the intraclass correlation coefficient (ICC) (Van Dongen et al. 2004; Tucker et al. 2007). For a given measure, total observed variance was partitioned into between‐ and within‐subject variances after correcting for known sources of variability. The ICC was estimated as ICC = *σ*^2^_BS_/(*σ*^2^_BS_ + *σ*^2^_WS_), where *σ*^2^_BS_ and *σ*^2^_WS_ are the between‐ and within‐subject variances, respectively. The ICC therefore reflects the proportion of overall variability in the data that is attributed to interindividual differences and can be interpreted according to benchmark ranges as follows (Landis and Koch 1977): “fair” (0.2–0.4), “moderate” (0.4–0.6), “substantial” (0.6–0.8), and “almost perfect” (0.8–1.0). Statistical significance of the ICC was assessed by a Wald Z‐test of the between‐subject variance (Snijders and Bosker 2012).

Linear mixed models were implemented to estimate between‐ and within‐subject variances while controlling for known sources of variability (Van Dongen et al. 2004). The model included a normally distributed random intercept to represent between‐subject differences around a fixed intercept, a normally distributed random error for within‐subject differences, and incorporated fixed effect corrections for Visit (first vs. second laboratory visit) and Protocol (Protocol 1 vs. 2). The interaction between Visit and Protocol, as well as age and BMI did not improve goodness of fit of the model, and so these terms were excluded. Between‐ and within‐subject variances were assumed to be independent, and the linear mixed models were evaluated using the restricted maximum likelihood method. The model was also used to assess statistical significance of the fixed effects.

To evaluate whether individual differences in behavior and physiology during sleep deprivation were related to baseline differences when subjects were well rested, the analysis was repeated with a model that included daytime performance/physiology (i.e., across six PVT sessions, 4.5–14.5 h after wake) as a covariate. For those instances in which the baseline covariate was confounded by a fixed effect, that is, it was statistically significant for both baseline and sleep deprivation values, variance uniquely explained by the baseline covariate was obtained by expressing the total variance (between‐ plus within‐subject variance) of an intercept plus baseline covariate‐only model as a percentage of the total variance of an intercept‐only model. Statistical significance was assessed by a likelihood ratio test between the two models. In another analysis, EEG‐based measures were evaluated with a model that included electrode impedance as a covariate, in order to exclude electrode impedance as a confounder. Statistical analyses were performed using SPSS software (IBM Corp., New York, NY), and *P *<**0.05 was considered statistically significant.

## Results

### Individual differences in baseline sleep measures were reproducible across two study visits

**Figure 1.**
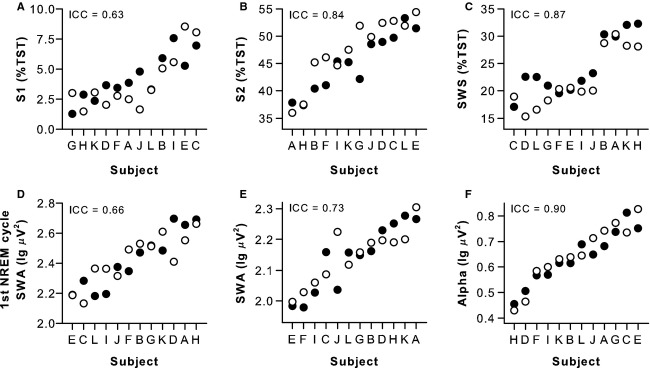
Individual differences in baseline sleep measures were stable across study visits. Sleep was assessed polysomnographically during two study visits spaced at least 2.5 months apart, with 8 h of time in bed for sleep scheduled at each participant's usual bedtime. Black circles show results for the first study visit, and open circles show results for the second study visit. Subjects (*n* = 12, A–L) are ranked from left to right according to their average response across study visits. Between‐subject differences in sleep staging results were reproducible for (A) Stage 1 sleep (S1), (B) Stage 2 sleep (S2), and (C) slow wave sleep (SWS), expressed as a percentage of total sleep time (TST). Individual differences in electroencephalogram (EEG) spectral power were stable across study visits for (D) slow wave activity (SWA) in the first NREM sleep cycle, (E) SWA during NREM sleep assessed across the entire sleep episode, and (F) alpha band activity during REM sleep. Results for EEG spectral analyses were obtained from the central derivation (i.e., average of C3–A2 and C4–A1). The intraclass correlation coefficient (ICC) is shown at the top left of each plot.

Twelve subjects participated in two laboratory studies separated in time by at least 2.5 months. In the month prior to each laboratory visit, within‐subject sleep quality and chronotype were similar, as determined by PSQI and MEQ scores, respectively (Table 1). Individual sleep behavior was also similar in the week before each study, as assessed by actigraphy‐estimated total sleep time, sleep efficiency, and bedtimes and wake times reported in subjects' sleep diaries. After being admitted to each laboratory study in the evening, participants were given 8 h of time in bed for sleep at their usual prestudy bedtime. Based on polysomnographically assessed sleep, individual differences in time spent in S1 sleep, S2 sleep, and SWS (as a proportion of total sleep time) were stable across study visits with ICC values ranging from “substantial” to “almost perfect” (Fig. 1A–C, z > 1.76, *P *<**0.04, ICC = 0.63–0.87). By comparison, there were no significant between‐subject differences in total sleep time, sleep latency, sleep efficiency, time spent in REM sleep, or wake after sleep onset (Wald Z‐test of between‐subject variances, *z* < 1.16, *P *>**0.12). Next, we evaluated whether EEG‐based markers of homeostatic sleep pressure were stable across study visits (Dijk et al. 1997; Roth et al. 1999). We found that individual differences in EEG spectral power were reproducible for SWA assessed during the first NREM cycle or across the entire sleep episode, as well as for alpha power during REM sleep (Fig. 1D–F, *z* > 1.83, *P *<**0.03, ICC = 0.66–0.90). Electrode impedance was not a significant covariate of any polysomnographically assessed sleep measure (F < 1.68, *P *>**0.21).

**Table 1.** Sleep behavior prior to study visits.

Measure	Visit 1	Visit 2	*t*	*P*
PSQI	2.3 ± 0.3	2.7 ± 0.5	−0.81	0.44
MEQ	50.2 ± 1.3	49.3 ± 1.5	0.59	0.57
Total sleep time (h)	7.7 ± 0.09	7.6 ± 0.06	−0.69	0.51
Sleep efficiency (%)	94.6 ± 0.8	94.8 ± 0.6	0.37	0.56
Bedtime (h)	00:38 ± 9 min	00:05 ± 14 min	1.46	0.18
Wake time (h)	08:45 ± 9 min	08:07 ± 15 min	1.81	0.10

Sleep behavior did not differ before study visits. Twelve subjects participated in two sleep deprivation studies spaced at least 2.5 months apart. Sleep quality and chronotype were assessed prior to each study visit using the Pittsburgh Sleep Quality Index (PSQI) and the Horne‐Östberg morningness–eveningness questionnaire (MEQ). Actigraphy monitoring was performed in the week prior to the study visit to estimate total sleep time and sleep efficiency. For each measure the mean ± SEM is shown. Values were compared between study visits using a paired *t*‐test.

### Individual differences in vigilance were reproducible across two exposures to sleep deprivation

**Figure 2.**
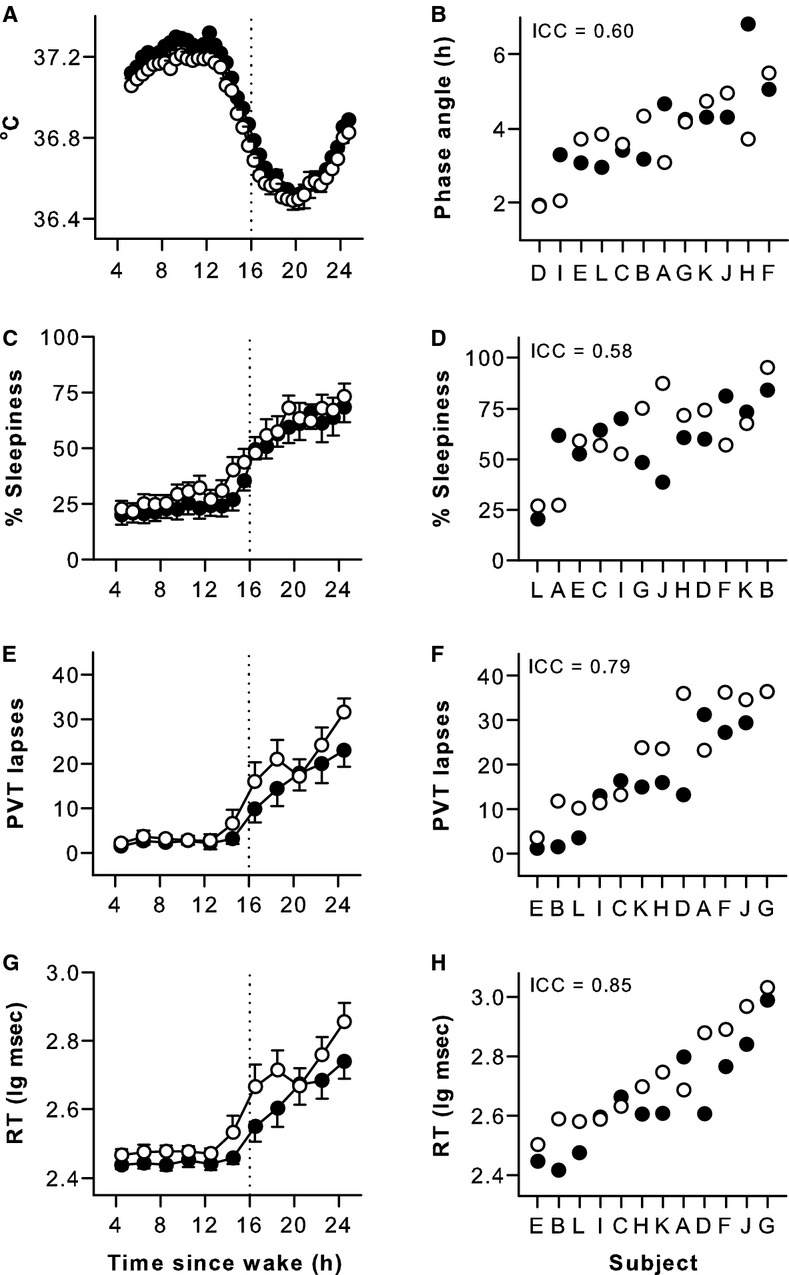
Individual differences in circadian phase, self‐rated sleepiness, and sustained attention were reproducible across repeated exposures to sleep deprivation. Subjects were kept awake for at least 26 h on two separate occasions. Based on the core body temperature rhythm (A), circadian phase was moderately stable across study visits, determined as the time difference in hours between the minimum of the fitted rhythm and habitual wake time (B). Self‐rated sleepiness increased after usual bedtime (C) and between‐subject differences during sleep deprivation were moderately stable (D). Sustained attention performance on the psychomotor vigilance task (PVT) became increasingly worse during prolonged wakefulness, and individual differences in performance were highly reproducible across exposures to sleep deprivation, as shown for PVT lapses (E, F) and PVT response times (RT) (G, H). Black circles show results for the first study visit and open circles show results for the second study visit. In panels A, C, E, and G, the mean ± SEM is shown. In panels B, D, F, and H, subjects (*n* = 12, A–L) are ranked from left to right according to their average response across study visits, and the intraclass correlation coefficient (ICC) is shown at the top left of each plot.

After baseline sleep, participants were kept awake for at least 26 consecutive hours. Across study visits, individual differences in circadian phase were moderately stable (ICC = 0.60), as shown for the core body temperature rhythm (Fig. 2A and B). As wakefulness was extended beyond habitual bedtime, self‐rated sleepiness increased monotonically, as did reaction time and lapses on the PVT (Fig. 2). Individual differences in self‐rated sleepiness were moderately reproducible across repeated exposures to sleep deprivation (Fig. 2C and D, ICC = 0.58), whereas between‐subject differences in PVT performance measures were highly stable (*Z *>**2.05, *P *<**0.020, ICC > 0.79) (Fig. 2E–H, Table 2). A small but significant difference in self‐reported sleepiness was found between protocols, with participants reporting lower levels of sleepiness during Protocol 2. In addition, performance on the PVT was worse, on average, during the second study visit (Table 2).

**Table 2.** Variance components analysis of behavioral and physiologic measures during sleep deprivation.

Measure	Variance analysis			Protocol		Study visit	
	*Z*	*P*	ICC	*F*	*P*	*F*	*P*
Self‐rated sleepiness	1.67	0.048*	0.58	11.8	0.01*	0.4	0.54
PVT lapses	2.05	0.020*	0.79	1.7	0.22	5.0	0.05*
PVT mean log RT	2.15	0.016*	0.85	3.1	0.11	9.5	0.01*
PERCLOS	2.19	0.014*	0.92	0.1	0.76	4.3	0.08
Blink rate	2.13	0.016*	0.84	3.3	0.10	0.4	0.54
Heart rate	2.05	0.020*	0.79	0.7	0.41	0.8	0.40
SDNN	2.17	0.015*	0.87	4.4	0.06	0.5	0.51
EEG delta power	1.93	0.027*	0.74	<0.01	0.89	1.0	0.35
EEG theta power	2.19	0.014*	0.88	<0.01	1.00	2.2	0.17
EEG alpha power	2.23	0.013*	0.91	<0.01	0.91	0.1	0.79
EEG beta power	2.07	0.019*	0.80	<0.01	0.97	<0.01	0.88

Individual differences in behavioral and physiologic measures during sleep deprivation were assessed in subjects who were kept awake for 26 h on two separate occasions. Variance components analysis was performed, with between‐ and within‐subject variance estimated using a linear mixed model with fixed effect corrections for Protocol (Protocol 1 vs. 2) and Visit (Visit 1 vs. 2). The intraclass correlation coefficient (ICC) is the between‐subject variance expressed as a proportion of total variance (between‐ plus within‐subject variance). ICC values range from 0 to 1, with values closer to 1 indicating greater stability in individual differences across study visits. Statistical significance of between‐subject variance and fixed effects were assessed using a Wald *Z*‐test and *F*‐test, respectively. Asterisks (*) highlight comparisons that were statistically significant (*P *<**0.05). PVT, psychomotor vigilance task; RT, response time; PERCLOS, percentage eyelid closure over the pupil over time; SDNN, standard deviation of normal‐to‐normal RR intervals; EEG, electroencephalogram.

### Individual differences in physiologic measures were reproducible across two exposures to sleep deprivation

**Figure 3.**
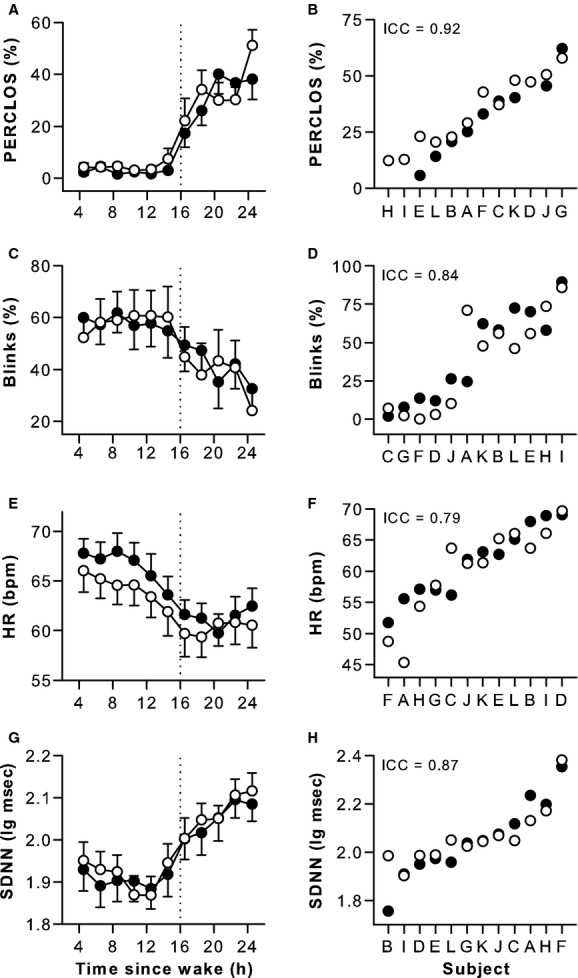
Individual differences in ocular and electrocardiogram measures during sleep deprivation were highly reproducible. As wakefulness was extended beyond usual bedtime, subjects closed their eyes a greater percentage of the time and blinked less frequently, and between‐subject differences were stable across exposures to sleep deprivation (A–D). During the usual hours of sleep, heart rate decreased and variability in heart rate increased, and differences in these measures were highly reproducible between subjects (E–H). Black circles show results for the first study visit, and open circles show results for the second study visit. In panels A, C, E, and G, the mean ± SEM is shown. In panels B, D, F, and H, subjects (*n* = 12, A–L) are ranked from left to right according to their average response across study visits, and the intraclass correlation coefficient (ICC) is shown at the top left of each plot. In three participants (D, H, and I), percentage eye closure was not assessed during the first study visit. PERCLOS, percentage eyelid closure over the pupil over time; HR, heart rate; bpm, beats per minute; SDNN, standard deviation of normal‐to‐normal RR intervals.

**Figure 4.**
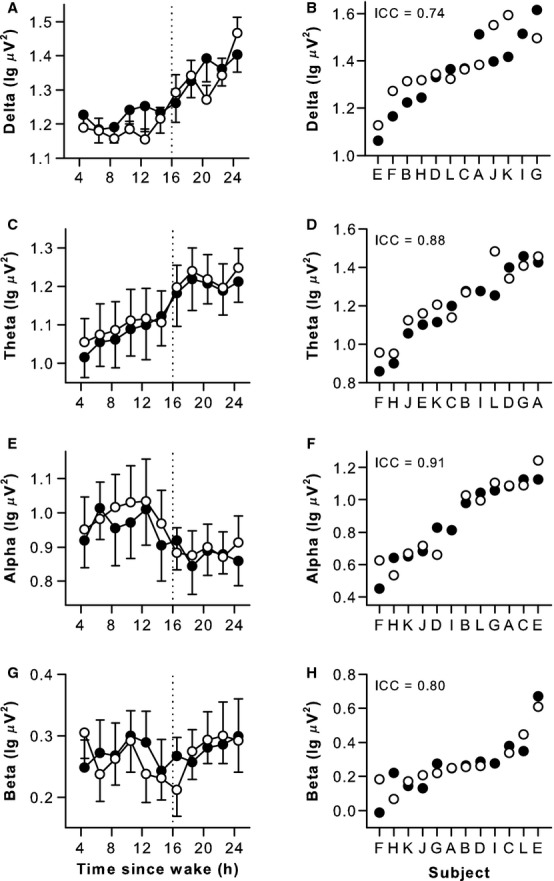
Individual differences in waking EEG spectral power during sleep deprivation were reproducible across study visits. During prolonged wakefulness, EEG spectral power in delta and theta frequency bands increased, and between‐subject differences were stable over repeated exposures (A–D). Alpha power decreased during the usual hours of sleep and beta power was lowest near bedtime. Individual differences in EEG alpha and beta activity were reproducible (E–H). Results are shown for the frontal EEG derivation. Black circles show results for the first study visit, and open circles show results for the second study visit. In panels A, C, E, and G, the mean ± SEM is shown. In panels B, D, F, and H, subjects (*n* = 12, A–L) are ranked from left to right according to their average response across study visits, and the intraclass correlation coefficient (ICC) is shown at the top left of each plot.

Similar to results for sustained attention performance, individual differences in ocular‐based metrics of sleepiness were highly stable and ICC values were in the “almost perfect” range (Fig. 3A–D, Table 2; ICC = 0.92 and 0.84). Subjects were also very consistent in their relative rankings for ocular measures, such that individuals who closed their eyes a greater percentage of the time during sleep deprivation blinked less frequently (Spearman's *rho* = −0.83, *P *=**0.001). Parallel results were observed for heart rate and its variability, which were negatively correlated (Spearman's *rho* = −0.92, *P *<**0.001), and highly reproducible across exposures to sleep deprivation (Fig. 3E–H, Table 2; ICC = 0.79 and 0.87). Consistent with prior work (Borbely et al. 1981; Cajochen et al. 1999; Finelli et al. 2000; Chua et al. 2012), EEG spectral power in the delta and theta frequency bands increased during prolonged wakefulness (Fig. 4A–D). In contrast, alpha power was lowest during the usual hours of sleep, and beta power reached its minimum level near bedtime (Fig. 4E–H). In each EEG frequency band examined, individual differences in spectral power were stable in response to sleep deprivation, with ICC values in the “substantial” to “almost perfect” range (Fig. 4, Table 2; ICC = 0.74–0.91). Subjects' relative rankings were variable across EEG frequency bands, however, with only spectral power in the alpha and beta bands exhibiting a significant correlation (Spearman's *rho* = 0.79, *P *=**0.002).

Since individual differences in some baseline sleep measures were reproducible, and behavioral and physiologic responses to sleep deprivation were stable across repeated exposures, we considered the possibility that these outcomes might be related. To assess this, we compared the relative rankings of subjects for sleep and wake measures using Spearman's correlation analysis (Table 3). Participants with a greater amount of S1 sleep at baseline performed better on the PVT during subsequent sleep deprivation, and displayed higher waking EEG spectral power in the spindle frequency range of the beta band. A greater amount of S2 sleep was also associated with greater spectral power in the low‐frequency beta band during sleep deprivation, but was not related to other physiologic measures or PVT performance. Slow wave sleep and SWA during baseline sleep did not show a significant correlation with any behavioral or physiologic measure taken during sleep deprivation. By comparison, participants who exhibited higher levels of alpha power during REM sleep rated themselves as feeling less sleepy during sleep deprivation, even though sustained attention performance and ocular measures of sleepiness were not correlated with self‐rated sleepiness or alpha activity. Subject ranks were very similar for alpha power assessed during REM sleep versus sleep deprivation, whereas SWA during baseline sleep did not correlate with EEG delta power during prolonged wakefulness (Table 3).

**Table 3.** Association between baseline sleep measures and behavioral and physiologic measures during prolonged wakefulness.

Waking measure	Sleep stage (% of TST)			EEG power	
	S1	S2	SWS	SWA	Alpha
Self‐rated sleepiness	−0.41	−0.39	0.29	0.22	−0.58*
PVT lapses	−0.68*	−0.30	0.02	0.26	−0.13
PVT mean log RT	−0.69*	−0.22	−0.05	0.23	−0.15
PERCLOS	−0.36	0.21	−0.35	0.15	0.22
Blink rate	0.18	−0.15	0.54	0.01	−0.28
Heart rate	0.36	0.43	−0.25	−0.09	−0.13
SDNN	−0.34	−0.32	−0.20	0.12	−0.06
EEG delta power	−0.25	−0.07	0.04	0.27	0.14
EEG theta power	−0.06	0.01	−0.25	0.41	0.25
EEG alpha power	0.55	0.43	−0.36	−0.18	0.85***
EEG beta power	0.73**	0.66*	−0.52	−0.36	0.52

For baseline sleep measures that showed stable between‐subject differences across study visits, we examined whether subject rankings were similar for behavioral and physiologic measures that were reproducible across repeated exposures to sleep deprivation. Results are shown for Stage 1 sleep (S1), Stage 2 sleep (S2), slow wave sleep (SWS), EEG spectral power for slow wave activity (SWA) in NREM sleep, and EEG spectral power in the alpha frequency band during REM sleep. For each subject and outcome measure, data were averaged across study visits and compared using Spearman's correlation analysis. Spearman's *rho* is shown for each comparison, with the level of statistical significance indicated by the asterisks: *P <**0.05; **P <**0.01; ***P <**0.001. PVT, psychomotor vigilance task; RT, response time; PERCLOS, percentage eyelid closure over the pupil over time; SDNN, standard deviation of normal‐to‐normal RR intervals; EEG, electroencephalogram.

### Baseline individual differences in behavioral and physiologic measures contributed substantially to differences observed during sleep deprivation

Next, we evaluated whether baseline differences in PVT performance and physiologic measures, assessed during the first 16 h of wakefulness, contributed significantly to observed between‐subject variability during sleep deprivation. With the exception of PERCLOS, all baseline measures contributed significantly to total variance for the same measures assessed during sleep deprivation (Table 4). Controlling for fixed effects, baseline individual differences in PVT response times, blink rate, heart rate and its variability, and EEG spectral power in theta, alpha, and beta frequency bands explained more than 50% of the variance in the sleep‐deprived state. After taking into account baseline measures as a covariate, between‐subject differences in PVT response times and lapses, and EEG spectral power in delta, theta, and beta bands did not reach statistical significance during sleep deprivation (*P *=**0.053–0.116). By comparison, self‐rated sleepiness, ocular and ECG‐derived measures, and EEG alpha activity showed significant stability across exposures to sleep deprivation, even after taking into account the contribution of baseline differences, with ICC values in the “substantial” to “almost perfect” range (ICC = 0.66–0.91).

**Table 4.** Variance components analysis of responses to sleep deprivation with baseline measures included as a covariate.

Measure	Baseline covariate			Variance analysis		
	*F*	*P*	%	*Z*	*P*	ICC
Self‐rated sleepiness	5.6	0.028^†^	18.1	1.80	0.036^#^	0.66
PVT lapses	10.3	0.005*	42.6	1.20	0.116	0.49
PVT mean log RT	9.9	0.006^†^	50.6	1.62	0.053	0.66
PERCLOS	<0.01	0.907	<0.01	1.96	0.025^#^	0.91
Blink rate	15.9	0.001*	52.8	1.74	0.041^#^	0.67
Heart rate	122.0	<0.001*	86.6	1.91	0.028^#^	0.72
SDNN	67.3	<0.001*	81.3	1.77	0.038^#^	0.66
EEG delta power	7.0	0.018*	24.0	1.60	0.055	0.60
EEG theta power	57.3	<0.001*	66.3	1.47	0.071	0.53
EEG alpha power	48.9	<0.001*	62.3	1.94	0.026^#^	0.76
EEG beta power	19.1	0.001*	50.6	1.36	0.087	0.49

Variance components analysis was performed to assess interindividual differences in responses to sleep deprivation, as described in Table 2, but this time taking into account baseline differences between subjects. Between‐ and within‐subject variances were estimated using a linear mixed model that included the corresponding baseline measure as a covariate, and with fixed effect corrections for protocol and study visit. The percentage of total variance explained by the same measure at baseline was estimated, with statistical significance assessed using an *F*‐test. Asterisks (*) indicate that the baseline covariate was statistically significant (*P *<**0.05), and daggers (†) indicate that the baseline covariate was statistically significant but confounded by a fixed effect. Hash marks (#) show between‐subject differences during sleep deprivation that were significant, after taking into account baseline individual differences in the same measures.

## Discussion

Here, we observed strong individual differences in behavioral and physiologic measures during prolonged wakefulness. For the first time, we demonstrate that ocular, ECG, and EEG‐derived measures show stable between‐subject differences across repeated exposures to sleep deprivation, as assessed by variance components analysis. A significant proportion of variance in physiologic measures during sleep deprivation was explained by baseline individual differences. Nonetheless, between‐subject differences in eye closures, heart rate and its variability, and EEG alpha activity were significant, even after taking into account baseline individual differences in these measures. These findings raise the possibility that physiologic responses to sleep loss are trait‐like, similar to cognitive vulnerability to total sleep deprivation.

### Between‐subject differences in sustained attention are highly reproducible

Consistent with previous work (Van Dongen et al. 2004; Rupp et al. 2012), we showed that individual differences in PVT performance were stable across repeated exposures to sleep loss. In these studies and our own, baseline individual differences in PVT performance contributed significantly to between‐subject variance in performance during sleep deprivation, and this was reflected by a decrease in the ICC value when adjusting for baseline as a covariate. We recently showed that individual differences in baseline PVT performance associated with decrements in vigilance during total sleep deprivation (Chua et al. 2014). In that study, small baseline differences appeared to be amplified during sleep deprivation, leading to large between‐subject differences in PVT performance. It should be emphasized, however, that baseline differences do not fully explain between‐subject differences in performance during sleep deprivation, as shown in earlier studies for PVT lapses and other behavioral measures (Van Dongen et al. 2004; Rupp et al. 2012). It was recently demonstrated that PVT performance during sleep deprivation has a strong heritable component (Kuna et al. 2012); however, genes that reliably associate with neurobehavioral responses to sleep loss have yet to be identified.

### Ocular and ECG‐derived measures show stable between‐subject responses to total sleep deprivation

We found that PERCLOS was the most stable measure across repeated exposures to sleep deprivation (ICC = 0.91). PERCLOS is perhaps the best characterized ocular measure of drowsiness and has been shown to correlate with PVT lapses during sleep deprivation (Dinges et al. 1998; Chua et al. 2012), as well as measures of simulated driving performance (Wierwille et al. 1996). In contrast to all other measures examined, baseline individual differences in PERCLOS did not contribute to between‐subject differences during sleep deprivation. This could be due to a floor effect, as long‐duration eye closure events were infrequent during baseline, thus compressing between‐subject variability. Similar to PERCLOS, between‐subject differences in blinking behavior were stable across study visits, even after taking into account differences in blink rate at baseline.

Similar to ocular measures, we found that heart rate and its variability exhibited strong individual differences during extended wakefulness. In previous studies, it was shown that several measures of daytime heart rate variability show stable between‐subject differences when studied in healthy participants under rested conditions (Marks and Lightfoot 1999; Sandercock et al. 2005; Pinna et al. 2007). In addition, several studies of twins have shown that individual differences in ECG‐derived measures are explained in part by genetic factors (Voss et al. 1996; Russell et al. 1998; Uusitalo et al. 2007). Our finding that between‐subject differences in ECG‐derived measures were stable during sleep deprivation could reflect individual differences in autonomic nervous system responses, as cardiac activity is modulated by sleep loss (Spiegel et al. 1999; Zhong et al. 2005; Mullington et al. 2009). Our findings suggest that changes in sympathovagal balance that occur during a night of sleep deprivation are stable across individuals, which might have implications for cardiovascular disease risk in persons who regularly work extended hours or night shifts.

### Between‐subject differences in EEG alpha activity are highly reproducible during sleep and in response to sleep deprivation

The waking EEG is thought to be one of the most heritable physiologic signals, based on numerous studies in monozygotic and dizygotic twin pairs (Stassen et al. 1987; van Beijsterveldt et al. 1996). Moreover, between‐subject differences in spectral power are stable across weeks or months, assessed across the major EEG frequency bands (Gasser et al. 1985; Pollock et al. 1991; Salinsky et al. 1991). In particular, strong individual differences in EEG alpha activity are observed when assessed in rested participants who have their eyes closed. Here, we demonstrate that between‐subject differences in delta, theta, alpha, and beta activity are reproducible across repeated exposures to sleep deprivation, assessed when participants were taking the PVT. After adjusting for individual differences at baseline, however, only EEG alpha power exhibited significant individual differences in response to sleep deprivation. These findings are consistent with a previous study in which the most reproducible EEG measure during total sleep deprivation was a decrease in high‐frequency alpha power (Leproult et al. 2003). Together, the aforementioned studies indicate that EEG spectral power in the alpha frequency band exhibits stable individual differences during rested and sleep deprived states, presumably due to genetically determined differences in EEG‐generating mechanisms.

Similar to the waking state, individual differences in the EEG during sleep are thought to be highly heritable and stable within subjects over time (De Gennaro et al. 2005, 2008; Buckelmuller et al. 2006; Tucker et al. 2007; Ambrosius et al. 2008; Campbell and Feinberg 2009; Tarokh and Carskadon 2010; Tarokh et al. 2011). Interestingly, between‐subject differences in the sleep EEG are relatively stable across different wake‐sleep manipulations, for example, during baseline sleep or recovery sleep following extended wakefulness. Despite an increase in low‐frequency EEG activity and a reduction in power in the high‐frequency range during prolonged wakefulness, the topographical distribution of EEG power across the scalp in NREM sleep is largely invariant when compared to baseline sleep (Finelli et al. 2001; De Gennaro et al. 2005). Moreover, the topographical EEG pattern is subject‐specific and genetically determined, as demonstrated in twin studies (De Gennaro et al. 2008). Individual differences in polysomnography (PSG)‐assessed sleep parameters have also been shown to be stable over time, including sleep Stages 2 through 4 and REM sleep (Buckelmuller et al. 2006). Moreover, studies in twins have shown that NREM sleep stages are strongly influenced by genetic differences (Linkowski 1999; Kuna et al. 2012), which is further supported by studies conducted in genetically modified mice (Shiromani et al. 2004). In a recent study that used mixed model variance components analysis, it was found that nearly all clinical sleep parameters exhibit trait‐like stability (Tucker et al. 2007). Using a similar analysis approach, we observed significant between‐subject differences in time spent in S1, S2, and SWS, but not for total sleep time, sleep efficiency, sleep latency, time spent in REM sleep, or wakefulness after sleep onset. Differences between studies could be explained by time in bed allotted for sleep, as subjects were given 8 h of time in bed for sleep in the present study. By comparison, in the study by Tucker et al., subjects were given 12 h of time in bed for sleep, which would allow for a larger between‐subject spread in sleep duration and sleep staging parameters.

Similar to results for NREM sleep stages, we observed stable between‐subject differences in SWA during NREM sleep and for spectral power in the alpha frequency band during REM sleep. Individual differences in alpha activity were in the almost perfect range (ICC = 0.90), closely resembling results for waking alpha spectral power (ICC = 0.91). In fact, the relative ranks of subjects was nearly the same for alpha activity measured during REM sleep versus wakefulness (Spearman's rho = 0.85, *P *<**0.001), suggesting that individual differences in these measures share a common biological basis. In contrast, SWA during NREM sleep did not associate with EEG delta power during extended wakefulness. Notably, the only sleep parameter that exhibited stable between‐subject differences and also correlated (negatively) with PVT performance was the percentage of time that participants spent in Stage 1 sleep. Both Stage 1 sleep and decrements in PVT performance are heritable, as demonstrated in a recent study of monozygotic versus dizygotic twin pairs (Kuna et al. 2012); however, the relationship between baseline sleep parameters and PVT performance was not examined in that study. Additional studies are needed to establish whether trait‐like differences in sleep architecture contribute to differences in vigilance during the daytime or in response to prolonged wakefulness.

### Limitations

In the absence of genetic evidence, individual differences in a given outcome measure can only be considered trait‐like if they are significant, stable when measured across repeated exposures, and robust when manipulated experimentally (Van Dongen et al. 2005). While our results meet the first two criteria, we did not experimentally manipulate environmental factors (e.g., sleep history or caffeine intake) that can influence behavioral and physiologic responses to sleep deprivation. Rather, participants underwent the same research procedures for each exposure to sleep deprivation. As such, we do not claim that our results demonstrate trait‐like differential responses to sleep deprivation. Given that EEG and ECG‐derived measures are heritable, we consider it likely that physiologic responses to total sleep deprivation are in fact trait‐like, but this hypothesis requires further testing. Another limitation of the present study is that we did not separate the effects of circadian and homeostatic sleep mechanisms on behavioral and physiologic responses. We defined the sleep‐deprived state as the period between usual bedtime and wake time (i.e., over a period of 8 h), corresponding to when participants would normally be sleeping. During this time window, the circadian rhythm of sleep propensity increases, while homeostatic sleep pressure builds with increasing time spent awake (Czeisler and Gooley 2007). Together, these processes interact to promote sleepiness and degrade performance across the night, such that cognitive vulnerability reaches its highest levels near usual wake time. Several of the physiologic measures that we examined are regulated by the circadian system and sleep homeostat, including PERCLOS, blink rate, SDNN, and EEG spectral power (Chua et al. 2012). Since we examined the effects of a single night of sleep deprivation, we cannot distinguish the relative contributions of circadian and homeostatic processes on between‐subject differences in behavioral and physiologic measures. Our results are nonetheless relevant for situations in which a person “pulls an all‐nighter,” or occupations with long work hours and sleep deprivation, for example, resident physicians, in whom vigilance levels are impacted by the interaction of circadian phase and sleep deficiency (Anderson et al. 2012).

Although the ICC values that we observed for PVT performance and PSG‐assessed sleep parameters were consistent with previous studies, it should be noted that our sample size was relatively small. Also, only ethnic‐Chinese males within a narrow age range were enrolled in the present study. As such, we cannot rule out the possibility that our results would differ if we included more subjects with greater heterogeneity. It will therefore be important to establish whether our findings extend to more diverse groups of individuals who differ in age, sex, and health status. Also, in future studies it should be assessed whether metabolic responses to sleep deprivation and circadian misalignment are stable and reproducible, as this could explain why some individuals are especially prone to metabolic dysfunction and obesity in response to shift work.

## Conclusion

Similar to behavioral outcomes, individual differences in ocular, ECG, and EEG measures are stable in response to sleep deprivation. Between‐subject differences in PERCLOS, blink rate, heart rate and its variability, and spectral power in the EEG alpha frequency band are highly reproducible, even after adjusting for baseline individual differences in these measures. These results extend prior work examining trait‐like differences in cognitive responses to sleep deprivation. Together, these studies indicate that, for a given individual, the brain responds predictably to the challenge of sleep deprivation. However, the neurobiologic and genetic basis for individual differences in behavioral and physiologic responses to sleep deprivation remains to be determined.

## Acknowledgments

We thank Eric Fang, Wen‐Qi Tan, Jonathan Chua, Merryn Ang, Szeching Lee, Esther Peh, Hui‐Ning Lim, and Jia‐Jia Chua for their assistance in carrying out these studies; staff members at the SingHealth Investigational Medicine Unit for providing medical supervision; and Dr. Hans van Dongen for his help in implementing the variance components analyses.

## Conflict of Interest

None declared.
